# Cross-cultural adaptation and psychometric properties of the Brazilian version of the scale of oral health outcomes for 5-year-old children (SOHO-5)

**DOI:** 10.1186/1477-7525-11-16

**Published:** 2013-02-09

**Authors:** Jenny Abanto, Georgios Tsakos, Saul Martins Paiva, Daniela Goursand, Daniela Prócida Raggio, Marcelo Bönecker

**Affiliations:** 1 Department of Pediatric Dentistry and Orthodontics, Faculty of Dentistry, University of São Paulo, Av. Lineu Prestes 2227, São Paulo, SP 05508-000, Brazil; 2 Department of Epidemiology and Public Health, University College London, 1-19 Torrington Place, London, WC1E 6BT, UK; 3 Department of Pediatric Dentistry and Orthodontics, Faculty of Dentistry, Federal University of Minas Gerais, Av. Antônio Carlos 6627, Belo Horizonte, MG, 31270-901, Brazil

**Keywords:** Oral health, Quality of life, Preschool children, Parents, Validation

## Abstract

**Background:**

Most of the instruments available to measure the oral health-related quality of life (OHRQoL) in paediatric populations focus on older children, whereas parental reports are used for very young children. The scale of oral health outcomes for 5-year-old children (SOHO-5) assesses the OHRQoL of very young children through self-reports and parental proxy reports. We aimed to cross-culturally adapt the SOHO-5 to the Brazilian Portuguese language and to assess its reliability and validity.

**Findings:**

We tested the quality of the cross-cultural adaptation in 2 pilot studies with 40 children aged 5–6 years and their parents. The measurement was tested for reliability and validity on 193 children that attended the paediatric dental screening program at the University of São Paulo. The children were also clinically examined for dental caries. The internal consistency was demonstrated by a Cronbach's alpha coefficient of 0.90 for the children’s self-reports and 0.77 for the parental proxy reports. The test-retest reliability results, which were based on repeated administrations on 159 children, were excellent; the intraclass correlation coefficient was 0.98 for parental and 0.92 for child reports. In general, the construct validity was satisfactory and demonstrated consistent and strong associations between the SOHO-5 and different subjective global ratings of oral health, perceived dental treatment need and overall well-being in both the parental and children’s versions (p < 0.001). The SOHO-5 was also able to clearly discriminate between children with and without a history of dental caries (mean scores: 5.8 and 1.1, respectively; p < 0.001).

**Conclusion:**

The present study demonstrated that the SOHO-5 exhibits satisfactory psychometric properties and is applicable to 5- to 6-year-old children in Brazil.

## Findings

### Background

Different oral health-related quality of life (OHRQoL) measures have been developed for children older than 6 years [1-7]. For younger children, research related to OHRQoL measures is limited; however, there is some evidence that children aged 4–6 years can also reliably report their own HRQoL [8,9]. Four measures have been used for very young children [10-13]. Two of these measures [10,11] are based on parental proxy reports, although it is recognised that proxy and children’s self-reports measure different realities [14-17]. Another measure [12] uses only children’s self-reported parameters for children aged 8 years or older and parental proxy reports for younger children.

Recently, the scale of oral health outcomes for 5-year-old children (SOHO-5) [13] was developed to assess the OHRQoL in young children through both self- and parental reports. This measure has not yet been validated in any language except English. Therefore, we cross-culturally adapted the SOHO-5 to the Brazilian Portuguese language and tested its reliability and validity in 5- to 6-year-old children.

## Methods

The SOHO-5 consists of a child self-report and a parental report of the child’s oral health history. Both versions contain 7 items. For the child version, the report refers to difficulties eating, drinking, speaking, playing, sleeping, smiling (due to pain) and smiling (due to appearance). The answers are reported using a 3-point scale (no = 0, a little = 1 and a lot = 2) aided by an explanation card with appropriate faces. The items in the parental version include difficulty eating, difficulty playing, difficulty speaking, difficulty sleeping, avoiding smiling due to pain, avoiding smiling due to appearance and affected self-confidence. The answering options follow a 5-point scale (no = 0, a little = 1, moderate = 2, a lot = 3 and a great deal = 4). A response of “Don’t know” was not used in the self-administered parental version, as we opted for an interview-administered questionnaire. The SOHO-5 scores are calculated as the sum of response codes. A higher score denotes a greater degree of oral impacts on the children’s quality of life.

### Translation and cross-cultural adaptation

The SOHO-5 was translated and adapted according to published standard guidelines [18-21]. Two translations into Portuguese were made by two native Portuguese translators. A revision panel evaluated the translations and determined the conceptual and item equivalence. The consensus-translated version was pilot tested on twenty 5- to 6-year-old children and their parents. The panel developed a pilot version, which was translated back into English by two bilingual translators. The back-translated English consensus version was compared with the original English version to determine semantic equivalence.

Finally, the draft Brazilian version was pilot tested for a second time on a different convenience sample of twenty 5- to 6-year-old children and their parents. There were no changes regarding new suggestions or difficulties of comprehension, and the panel approved the final Brazilian Version of the SOHO-5.

### Assessment of validity and reliability

Data were collected from interviews with 193 5- to 6-year-old children and their parents, who were recruited from a paediatric dental screening program at the Faculty of Dentistry, University of São Paulo (USP). Children aged 1–9 years living in São Paulo city were eligible to participate in the screening programme. Children that had not received dental treatment in the last three months, had no systemic diseases and lived with their parents were eligible for inclusion. The study was approved by the USP Ethics Committee in Research, and the parents signed informed consent forms.

The child and one of the parents completed the SOHO-5 in face-to-face independent interviews. The interviews were conducted on the same day prior to the clinical examinations by four trained interviewers who were blind to the clinical findings. The children’s oral examinations referred to dental caries according to standard widely applied clinical criteria [22] and were conducted by two paediatric dentistry specialists who were calibrated prior to data collection (Kappa: 0.92 for intra- and 0.87 for inter-examiner reliability).

The children’s and parental questionnaires both contained global rating questions. For the children’s questionnaires, the following ratings were included: satisfaction with oral health (‘How happy are you with your teeth?; not happy = 2, a little happy = 1 and very happy = 0’) and presence of dental cavities (‘Do you have any holes in your teeth?; No = 0, Yes = 1’). For the parental questionnaires, the following ratings were included: proxy-rated oral health (‘How would you rate your child’s dental health?; excellent = 0, very good = 1, good = 2, fair = 3, poor = 4’), satisfaction with child’s oral health (‘How happy are you with your child’s dental health?; very happy = 0 to very unhappy = 4’), the child’s overall well-being (‘Do you think the overall well-being of your child is affected by the conditions of their teeth?; not at all = 0 to a great deal = 4’), and the child’s perceived dental treatment needs (‘Do you think your child needs any dental treatment because of the state (holes in teeth or pain) of his/her teeth?; no = 0, Yes = 1’).

### Data analysis

Internal consistency was assessed using Cronbach's alpha for the total score and the item-total score correlations. The test-retest reliability was assessed by calculating the intraclass correlation coefficient (ICC) for the SOHO-5 score using the data from 159 children and their parents who were interviewed for a second time 7–14 days after the first interview by the same interviewers. We tested construct validity through associations between the SOHO-5 scores and the global ratings using Spearman's correlation coefficients. Discriminant validity compared the SOHO-5 scores between the children with a history of caries and the children without a history of caries (dmft > 0 vs. dmft = 0) using Mann–Whitney tests.

## Results

Overall, 219 children and their parents were invited to participate in the study. Sixteen were excluded because they did not conform to the study criteria. Of the 203 eligible participants, 193 provided signed parental informed consent (response rate: 95.1%).

The sample (n = 193) consisted primarily of boys (54.9%) and 5-year-old children (58.5%), and 44.6% of the sample population had a history of dental caries. Most of the parental questionnaires were answered by mothers (91.2%). The parental SOHO-5 score ranged from 0 to 24, with a mean of 3.67 (standard deviation: 5.54). The child SOHO-5 score ranged from 0 to 12, with a mean of 2.45 (sd: 2.92). More than 64% of the parents and 68% of the children reported oral impacts (SOHO-5 score > 0).

The Cronbach's alpha coefficients were 0.77 and 0.90 for the children’s and parental versions, respectively (Tables 1 and 2), which indicated good internal consistency. For test-retest reliability, the ICCs were 0.92 and 0.98 for the total scores of the children’s and parental versions, respectively, which indicated excellent reproducibility (Tables 1 and 2). The construct validity showed that the SOHO-5 total score was associated significantly and in the expected direction with two global rating questions for children and the four respective questions for parental proxy reports (Tables 3 and 4). For both versions, children with a history of dental caries exhibited significantly higher SOHO-5 total and item scores compared with children with no history of dental caries (Tables 5 and 6).

**Table 1:** Reliability statistics for total score and items in the children’s self-reported version (n = 159)

	**Reliability**	
	**Children’s version**	
	**Cronbach’s alpha§**	Intraclass correlation coefficient (95% CI)*
**Total score**	0.77	0.92 (0.89-0.94)
Difficulty eating	0.45	0.80 (0.72-0.85)
Difficulty playing	0.33	0.86 (0.81-0.90)
Difficulty speaking	0.39	0.89 (0.85-0.92)
Avoiding smiling (due to appearance)	0.51	0.86 (0.81-0.90)
Avoiding smiling (due to pain)	0.52	0.77 (0.69-0.83)
Difficulty sleeping	0.48	0.85 (0.80-0.89)
Difficulty drinking	0.27	0.60 (0.46-0.71)

* Two-way random effects model: p < 0.001 for all values.

§ Internal consistency (Cronbach’s alpha) was performed for the total score (7 items together) and item-total correlations.

**Table 2:** Reliability statistics for the total score and items in the parental version (n = 159)

	**Reliability**	
	**Parental version**	
	**Cronbach’s alpha §**	Intraclass correlation coefficient (95% CI) *
**Total score**	0.90	0.98 (0.97-0.99)
Difficulty eating	0.50	0.92 (0.89-0.94)
Difficulty playing	0.29	0.89 (0.85-0.92)
Difficulty speaking	0.44	0.96 (0.95-0.97)
Avoiding smiling (due to appearance)	0.43	0.93 (0.91-0.95)
Avoiding smiling (due to pain)	0.43	0.96 (0.95-0.97)
Difficulty sleeping	0.50	0.97 (0.96-0.98)
Affected self-confidence	0.42	0.87 (0.82-0.91)

* Two-way random effects model: p < 0.001 for all values;

§ Internal consistency (Cronbach’s alpha) was performed for the total score (7 items together) and item-total correlations.

**Table 3:** Construct validity for the children’s version (n = 193)

	**Satisfaction with oral health**		**Presence of dental cavities**	
	r*	**p-value**	r*	**p-value**
**Total score**	0.505	<0.001	0.527	<0.001
Difficulty eating	0.287	<0.001	0.426	<0.001
Difficulty drinking	0.210	0.003	0.260	<0.001
Difficulty speaking	0.366	<0.001	0.271	<0.001
Difficulty playing	0.310	<0.001	0.290	<0.001
Difficulty sleeping	0.455	<0.001	0.452	<0.001
Avoiding smiling (due to pain)	0.357	<0.001	0.339	<0.001
Avoiding smiling (due to appearance)	0.420	<0.001	0.260	<0.001

*Spearman's rank correlation coefficient

**Table 4:** Construct validity for the parental version (n = 193)

	**Proxy-rated oral health**		**Satisfaction with child’s oral health**		**Child’s perceived dental treatment need**		**Child’s overall well-being affected**	
	r*	**p-value**	r*	**p-value**	r*	**p-value**	r*	**p-value**
**Total score**	0.678	<0.001	0.677	<0.001	0.510	<0.001	0.600	<0.001
Difficulty eating	0.621	<0.001	0.619	<0.001	0.368	<0.001	0.546	<0.001
Difficulty speaking	0.310	<0.001	0.301	<0.001	0.170	0.018	0.286	<0.001
Difficulty playing	0.386	<0.001	0.333	<0.001	0.274	<0.001	0.451	<0.001
Difficulty sleeping	0.544	<0.001	0.522	<0.001	0.448	<0.001	0.499	<0.001
Avoiding smiling (due to appearance)	0.466	<0.001	0.431	<0.001	0.340	<0.001	0.416	<0.001
Avoiding smiling (due to pain)	0.613	<0.001	0.518	<0.001	0.369	<0.001	0.544	<0.001
Affected self-confidence	0.346	<0.001	0.267	<0.001	0.275	<0.001	0.397	<0.001

*Spearman's correlation coefficient

**Table 5:** Discriminant validity for the child version

	**Without caries experience (n = 107)**		**With caries experience (n = 86)**		p-value*
	**Mean (SD)**	**Median**	**Mean (SD)**	**Median**	
**Total score**	1.20 (2.06)	0.00	3.45 (3.12)	2.00	<0.001
Difficulty eating	0.35 (0.70)	0.00	0.91 (0.75)	1.00	<0.001
Difficulty drinking	0.09 (0.40)	0.00	0.25 (0.50)	0.00	0.002
Difficulty speaking	0.07 (0.26)	0.00	0.22 (0.56)	0.00	0.047
Difficulty playing	0.06 (0.24)	0.00	0.33 (0.66)	0.00	0.001
Difficulty sleeping	0.15 (0.45)	0.00	0.62 (0.80)	0.00	<0.001
Avoiding smiling (due to pain)	0.13 (0.34)	0.00	0.50 (0.78)	0.00	0.001
Avoiding smiling (due to appearance)	0.35 (0.63)	0.00	0.63 (0.82)	0.00	0.018

*Mann–Whitney test.

**Table 6:** Discriminant validity for the parental version

	**Without caries experience (n = 107)**		**With caries experience (n = 86)**		p-value*
	**Mean (SD)**	**Median**	**Mean (SD)**	**Median**	
Total score	1.06 (1.65)	0.50	5.78 (6.59)	4.00	<0.001
Difficulty eating	0.35 (0.61)	0.00	1.47 (1.41)	1.00	<0.001
Difficulty speaking	0.08 (0.32)	0.00	0.47 (0.97)	0.00	0.002
Difficulty playing	0.01 (0.11)	0.00	0.53 (1.16)	0.00	<0.001
Difficulty sleeping	0.15 (0.47)	0.00	1.21 (1.47)	1.00	<0.001
Avoiding smiling (due to appearance)	0.20 (0.43)	0.00	0.79 (1.26)	0.00	0.001
Avoiding smiling (due to pain)	0.05 (0.21)	0.00	0.73 (1.22)	0.00	<0.001
Affected self-confidence	0.22 (0.52)	0.00	0.58 (1.13)	0.00	0.048

* Mann–Whitney test.

## Discussion

This study cross-culturally adapted and successfully validated the SOHO-5 for use among Brazilian children and their parents. It also demonstrated that 5- to 6-year-old children in Brazil are capable of providing their own perceptions concerning their OHRQoL, and studies should no longer depend solely on parental proxy reports. Many subjects in the sample population (90.3%) were able to understand the SOHO-5 and respond appropriately to the questions, independently of the age or sex of the children.

In addition to a meticulous translation, we employed a pre-test phase, which is important for identifying potential problems with the questionnaire content, such as misunderstandings about the intended meaning of the items and their clarity. The results showed semantic equivalence between the English and Brazilian Portuguese language versions of the SOHO-5.

The psychometric properties of the SOHO-5 were satisfactory and provided strong support for its reliability and validity. The reliability of the SOHO-5 was established for both internal and test-retest consistency, and its validity was evident in the consistent and strong associations with different subjective global ratings of oral health, perceived dental treatment need and overall well-being in both the parental and children’s versions. The measure also demonstrated discriminant validity between clinical groups according to their caries history.

Despite the inclusive nature of the screening programme, the sample was not representative of the general population of 5- to 6-year-old children. Therefore, the prevalence of oral impacts in the general population may be different. Because our sample consisted of children that had already sought dental treatment in the screening program, the oral impacts among children with dental caries in the general population could be even higher because children may not seek or have access to dental care. However, the dental caries history in our sample (44.6%) was similar to that of the general population of 5-year-old children in Brazil (56.4%) [23].

We acknowledge that 5- and 6-year-old children are not developmentally identical. While the original SOHO-5 study referred to 5-year-old children, an older child may comprehend and answer the questions more easily due to more advanced cognitive development [24]. Our study, which included children primarily of middle and lower socioeconomic status with a low proportion of affluent children, would have benefited from a more balanced sample in relation to socioeconomic position. Finally, the paper focused on validating the SOHO-5 with clinical determinants accounting for the potential confounding effect of other factors, which would require a larger and more representative sample and is therefore a future priority.

## Conclusion

This study provides strong evidence supporting the reliability and validity of the Brazilian SOHO-5 to be used as an OHRQoL measure for 5- to 6-year-old Brazilian children.

## Competing interest

The authors declare that they have no competing interests.

## Authors’ contributions

JA was responsible for the acquisition of data, assisted in the analysis and interpretation of data and drafted the manuscript; GT was responsible for the conception and design of the study and critical manuscript review; SMP performed the design of the study, acquisition of data (Revision Panel), and helped with the statistical analysis and critical manuscript review; DPR was responsible for the acquisition of data (Revision Panel) and critical manuscript review; PC performed the acquisition of data and critical manuscript review; DG helped with the statistical analysis, interpretation of data and critical review; MB was responsible for the conception and design of the study, acquisition of data (Revision Panel) and critical manuscript review. All the authors read and approved the final manuscript.
